# A sentinel survey in remote Western Thailand indicates that school-aged children and reproductive-aged women of the Indigenous Pwo Karen community are iodine sufficient

**DOI:** 10.1017/S0007114524003325

**Published:** 2025-02-14

**Authors:** Pattamaporn Joompa, Pornpan Sukboon, Werner Schultink, Michael B. Zimmermann, Sueppong Gowachirapant

**Affiliations:** 1 Institute of Nutrition, Mahidol University, Salaya, Thailand; 2 Iodine Global Network, Orleans, ON, Canada; 3 MRC Translational Immune Discovery Unit, MRC Weatherall Institute of Molecular Medicine, University of Oxford, John Radcliffe Hospital, Oxford, UK

**Keywords:** Iodine status, Sentinel survey, Indigenous people, Urinary iodine concentration, Iodine content in salt, School-aged children, Reproductive-aged women

## Abstract

Indigenous peoples are often not routinely included in iodine programmes because of language barriers and remote access and may thus be at higher risk of iodine deficiency disorders, which could adversely impact their quality of life. We conducted this cross-sectional study in the remote Pwo Karen community of Thailand to determine the urinary iodine concentration of school-aged children and women of reproductive age and investigate the iodine content in household salt. We measured urinary iodine concentration in spot urine samples from healthy school-aged children and women of reproductive age, administered a questionnaire, estimated daily iodine intake and collected household salt samples to determine salt iodine concentration. The median urinary iodine concentration (range) of school-aged children (*n* 170) was 192 (136–263) µg/l, which was significantly higher than women of reproductive age (*n* 306) (147 (89–233) µg/l) (*P* < 0·001). The estimated daily iodine intake in school-aged children and women of reproductive age were 135 and 195 μg/d, respectively. The median (range) iodine concentration in rock and granulated salts consumed in the households were 2·32 (0·52–3·19) and 26·64 (20·86–31·01) ppm, respectively. Surprisingly, the use of iodised salt and the frequency of seafood consumption were NS predictors of urinary iodine concentration in these two groups. Our data suggest that school children and women of the Pwo Karen community have sufficient iodine intake, indicating the Thai salt iodisation programme is effectively reaching even this isolated Indigenous community. Sentinel surveys of remote vulnerable populations can be a useful tool in national iodine programmes to ensure that programme coverage is truly universal.

Iodine is an essential micronutrient involved in metabolism, growth and brain development in animals and humans^(1)^. Iodine deficiency causes a broad spectrum of adverse effects, including goitre, infertility and an increased risk of cretinism or thyroid cancer. Inadequate iodine, which is required for thyroid hormone production, affects thyroid hormone production, termed iodine deficiency disorders^(1–3)^. Iodine deficiency is a global public health problem affecting all groups of people, especially women more than men. Although severe endemic iodine deficiency has largely disappeared in most parts of the world, mild-to-moderate iodine deficiency is still widespread globally, especially in Asia^(4)^. To combat iodine deficiency, salt iodisation is recommended as the most cost-effective way of delivering iodine to the target population^(5)^. The WHO recommends iodised salt consumed in the household should contain 15–40 ppm of iodine^(2)^.

In Thailand, the iodine surveillance prevention and control programme has been operated continuously since 1989. Part of the strategy to drive this matter is to develop data management and research. Accordingly, salt iodisation and iodine supplementation in vulnerable groups such as pregnant women and children living in remote areas are the most common strategies^(6)^. The iodine status and effects of iodine supplementation in these populations are therefore continuously monitored^(7–10)^. From the surveillance data (2015–2016) of iodine status collected by the Bureau of Nutrition, Department of Health, Thailand, the median urinary iodine concentration (UIC) among children aged between 3 and 5 years and the elderly groups indicated an adequate iodine intake. In addition, the urinary iodine status among Thai pregnant women during 2015–2020 indicates an optimal iodine status^(11)^. However, the iodine status among SAC (6–12 years) and women of reproductive age (WRA) (15–49 years) has not been elucidated. In recent years, the quality of iodised edible salt in Thai households was estimated by a random inspection. The result showed that more than four-fifths of Thai households consumed adequate iodine content in salt (20–40 ppm)^(11)^. Although surveillance of Thai people’s iodine status indicated appropriate daily intake of iodine in children aged 3–5 years old, elderly and pregnant women, the iodine status of Indigenous people living in Thailand has not been specifically revealed. Indigenous people are one of the most marginalised people in many societies in terms of being excluded from access to socioeconomic rights, and this is especially the case with healthcare^(11)^. Most countries have inaccurate or no published statistical data for these people. Hence, systematic information on health, morbidity and mortality is rare and fragmented^(12)^.

To reach the iodine deficiency disorder surveillance programmes in Indigenous people, this study thus aims to determine the iodine status of Indigenous communities of two priority population subgroups, including SAC and WRA, and to investigate the iodine content in household salt. In addition, The results can also be considered and strengthened for the universal salt iodisation programme in the country, which can be described as covering Indigenous peoples.

## Methods

### Study design and study site

The cross-sectional study was conducted in Laiwo Subdistrict, Sangkhlaburi District, Kanchanaburi Province, Thailand. This subdistrict is located in Thungyai Naresuan National Wildlife Sanctuary adjacent to the Myanmar border. Access to this community is only by four-wheel drive vehicle or motorcycle. There are six villages with 1352 households in Laiwo Subdistrict. The population is 6183 inhabitants – 3283 males and 2900 females^(13)^. Due to a limitation in transportation and accessibility, only three villages were purposively collected for data collection, including Sanephong, Kongmongta and Koh Sadueng.

### Participants

The participants were divided into two groups: school-aged children (SAC) and women of reproductive age (WRA). We aimed to recruit a sample of 300 SAC and 300 WRA. This study was conducted according to the guidelines laid down in the Declaration of Helsinki, and all procedures involving human subjects were approved by the Center of Ethical Reinforcement for Research, Mahidol University (MUCIRB 2022/178·3006). Written informed consent was obtained from all subjects. The study protocol was registered on the ClinicalTrials.gov platform at https://clinicaltrials.gov/ (identifier: NCT05920603). After receiving the name list of villagers in each village, the sample quota in each selected village was estimated using a proportional selection technique. The inclusion and exclusion criteria were considered to recruit eligible participants in each village for the study. Inclusion criteria were (1) healthy SAC (6–12 years old) or healthy WRA (15–49 years old) who had not been diagnosed by a doctor having serious medical conditions, (2) living in the study area and (3) willing and able to give informed consent for participation in the trial. Exclusion criteria were (1) pregnant women, (2) breastfeeding women and (3) people who have moved out from the village. A simple random sampling using a lottery method was used for participant selection. For the SAC, it was found that the actual total of eligible children (*n* 219) was less than the required number, so all of them were invited.

### Study procedure

The sample collection period was from April to July 2023. The core research team presented the project at the monthly village meetings of all three villages and at Ban Kongmongta Subdistrict Health Promotion Hospital to invite interested local people to join the research team in each village. The local people who were fluent in reading and writing Thai and could translate between Karen and Thai were selected to be local researchers. After selection, three males and twenty-three females were selected to participate as local researchers and were given a clear explanation of the project details. Most of the local researchers had experience in data collection in their villages. Then, all local researchers attended training from the core research team on (1) how to complete the general information questionnaire, (2) how to collect urine samples and (3) how to collect salt samples.

### Data collection and analysis

*Questionnaire.* The WRA and guardians of SAC completed the questionnaire that included the following: (1) age of participants; (2) weight and height of participants; (3) perception about iodised salt; (4) intent to purchase iodised salt for household consumption; (5) whether salt was used more as a seasoning or a condiment in the household; (6) whether more salt or more fish sauce was used in the household (these two condiments are widely used in Thailand); and (7) frequency of household seafood consumption per week: never, 1–2 times, 3–4 times, or over 4 times.

*Urinary iodine concentration*. Spot urine samples were collected from eligible participants (SAC and WRA) at home in the morning using a small plastic bottle and zip-lock plastic bag. The urine samples were transported on ice to the Institute of Nutrition, Mahidol University, aliquoted into microcentrifuge tubes and stored at –20ºC until analysis. At the Institute of Nutrition Mahidol University, the iodine concentration in urine was measured in duplicate using the Pino modification of the Sandell–Kolthoff reaction^(14)^.

*Iodine content in salt.* Participants were asked for two tablespoons of salt commonly used in their households. The salt samples were transported to the Institute of Nutrition Mahidol University and stored under dry and shielded from light conditions until analysis. The iodine content in salt was measured using the iodometric titration technique^(15)^.

*Daily iodine intake.* UIC and body weight were used to estimate iodine intake through the formula of the US Institute of Medicine^(16)^:

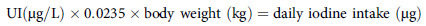




### Data and statistical analysis

Data analysis was performed using the SPSS version 21 statistical package (SPSS Corp.). The Kolmogorov–Smirnov test was used to assess normal data distribution. It was reported as the mean ± sd, while nonparametric values were reported as the median with interquartile range. The Mann–Whitney *U* test was used to test the UIC difference between SAC and WRA. To determine the association between the selected sociodemographic predictors and UIC, a binary logistic regression was conducted. Statistical significance was considered for *P* < 0·05.

## Results

### Characteristics of participants

A total of 170 SAC and 306 WRA were recruited from the three villages. The SAC comprised a similar proportion of gender (eighty-four males and eighty-six females), with a mean age of 9·3 ± 2·0 years, while the WRA had a mean age of 32·5 ± 10·7 years. The mean weights of SAC and WRA were 30·2 ± 12·2 kg and 56·4 ± 12·0 kg, respectively. The mean heights of SAC and WRA were 128·1 ± 18·3 cm and 153·9 ± 6·2 cm, respectively. The BMI of WRA was 23·85 ± 5·1 kg/m^2^. Regarding perception about iodised salt, most of SAC (62·9 %) and WRA (74·8 %) were aware of iodised salt. Regarding the households information collected from WRA and guardians of SAC, there was little difference in intention to buy or not to buy iodised salt for household consumption in both groups. The majority of households of both participant groups used salts as a seasoning (SAC, 99·4 %; WRA, 99·3 %) and consumed salt more than fish sauce (SAC, 98·2 %; WRA, 96·7 %). About two-thirds of both participant groups consumed seafood 1–2 times per week (SAC, 57·1 %; WRA 63·1 %) (Table 1).

**Table 1.** Characteristics of school-aged children (SAC) and women of reproductive age (WRA)

Variables	SAC *n* 170 (%)		WRA *n* 306 (%)	
	Mean	sd	Mean	sd
Age, y	9·3	2·0	32·5	10·7
Child’s gender				
Male:female, n:n	84:86			
Weight, kg	30·2	12·2	56·4	12·0
Height, cm	128·1	18·3	153·9	6·2
BMI, kg/m^2^	–		23·85	5·1
Perception about iodised salt, %				
Yes	62·9		74·8	
No	37·1		25·2	
Intent to purchase iodised salt, %				
Yes	44·1		43·1	
No	40·0		42·8	
Do not know	15·9		14·1	
Greater salt consumption for, %				
Cooking	99·4		99·3	
Condiment	0·6		0·7	
Greater consumption of, %				
Salt	98·2		96·7	
Fish sauce	1·8		3·3	
Seafood consumption per week, %				
Never	42·4		29·1	
1–2 times	57·1		63·1	
3–4 times	0·6		5·9	
Over 4 times	0		2·0	

### Iodine content of salt consumed by the participating household

A total of 370 salt samples (162 rock salt and 208 granulated salt) were obtained from 280 households (91·0 %). Among 280 households, households that consumed two types of salts, only rock salt and only granulated salt were 90 (32·1 %), 72 (25·7 %) and 118 (42·1 %), respectively. The median iodine content with interquartile range in rock and granulated salts was 2·32 (0·52–3·19) ppm and 26·64 (20·86–31·01) ppm, respectively. Most rock salt (99·4 %) had an iodine content of less than 15 ppm, while most granulated salt (75·0 %) had adequate iodine content of 15–40 ppm. In other words, rock salt is a non-iodised salt, whereas granulated salt is an iodised salt (Table 2).

**Table 2.** Iodine content in household salt (*n* 370)

Type of salt	Iodine content*						*n*
	< 15 ppm		15–40 ppm		> 40 ppm		
	Row %	Column %	Row %	Column %	Row %	Column %	
Rock	99·4	84·3	0·6	0·6	0		162
Granule	14·4	15·7	75·0	99·7	10·6	100·0	208
*n*	191		157		22		370

* Number of salt samples (row %, column %).

### Urinary iodine concentration

**Fig. 1.**
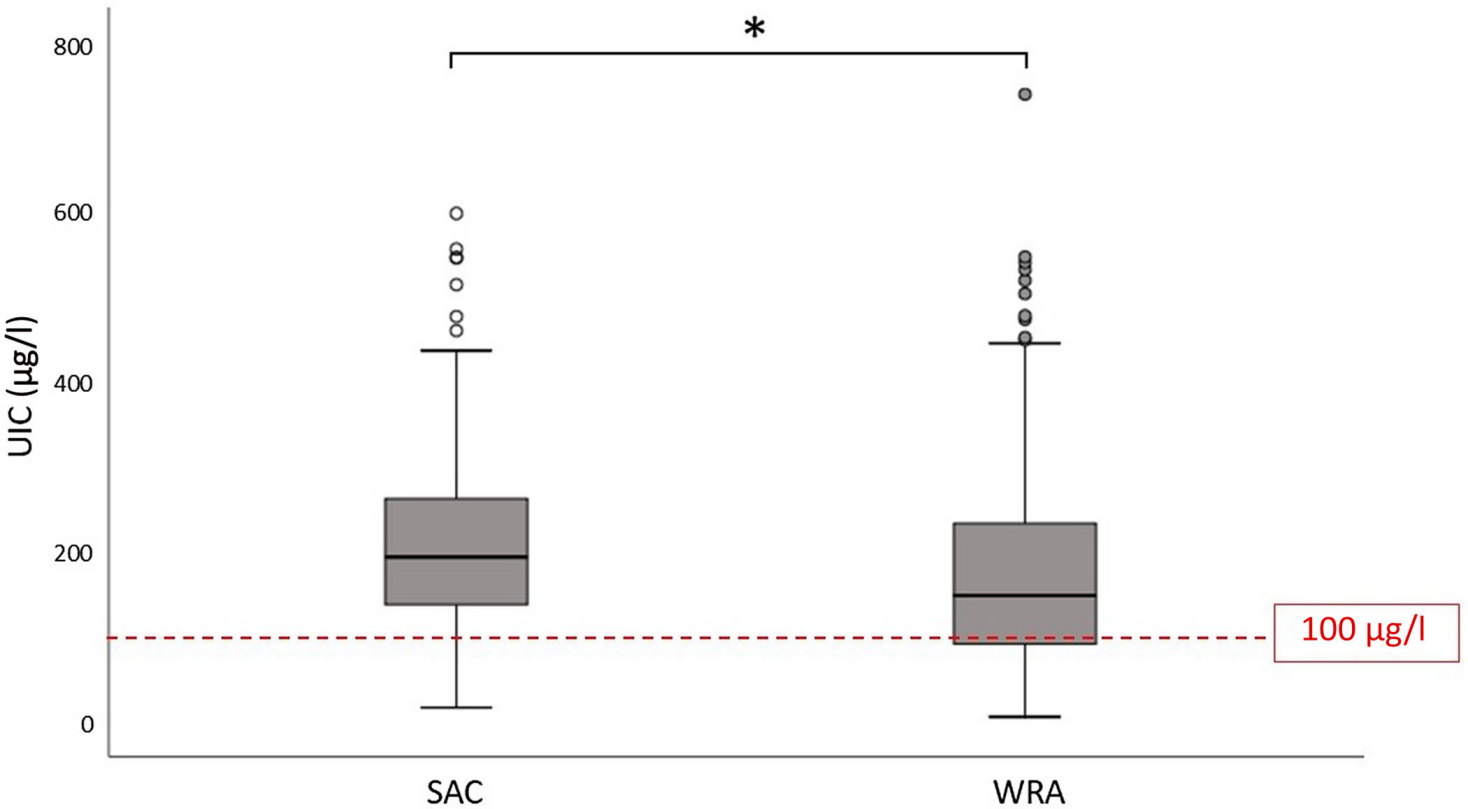
Urinary iodine concentration (UIC) of school-aged children (SAC) and women of reproductive age (WRA). Horizontal lines and boxes represent the median and the interquartile range, respectively. Whiskers indicate sd. The stippled horizontal line represents the epidemiological criteria for assessing adequate iodine intake based on the median UIC (2). *(*P* < 0·001) indicates a significant difference in UIC (Mann–Whitney *U* test).

The median UIC with interquartile range of SAC (192 (136–263) µg/l) was significantly higher than WRA (147 (89–233) µg/l) (*P* < 0·001) (Fig. 1). According to WHO criteria^(2)^, 40·0 % of SAC and 37·6 % WRA had a UIC that fell between 100–199 μg/l, 1·8 % of SAC and 8·5 % of WRA had a UIC < 50 μg/l, while 20·0 % of SAC and 14·1 % of WRA had a UIC ≥ 300 μg/l (Table 3). The median UIC of 192 μg/l in SAC with a mean age of 9·3 years and a mean body weight of 30·2 kg was related to a median iodine intake of approximately 135 μg/d. While among WRA with a mean age of 32·5 years and a mean body weight of 56·4 kg, their median UIC of 147 µg/l corresponded to a median iodine intake of approximately 195 μg/d.

**Table 3.** Median (IQR) urinary iodine concentration in school-aged children (SAC) and women of reproductive age (WRA)

Median urinary iodine concentration (μg/l)*	Frequency			
	SAC (*n* 170)		WRA (*n* 306)	
	*n*	%	*n*	%
< 20	1	0·6	3	1·0
20–49	2	1·2	23	7·5
50–99	18	11·0	61	19·9
100–199	68	40·0	115	37·6
200–299	47	27·7	61	19·9
≥ 300	34	20·0	43	14·1

* WHO (2007) criteria for iodine sufficiency in school-aged children and women with reproductive age^(2)^.

### Association of sociodemographic variables and urinary iodine concentration

The OR for having UIC < 100 µg/l, according to household use of granulated salt, which is an iodised salt, and the frequency of seafood consumption per week were determined. Accordingly, SAC whose households did not use iodised salt (OR 1·175, 95 % CI 0·358, 3·854, *P* = 0·791) and did not consume seafood in the past week (OR 1·024, 95 % CI 0·407, 2·578, *P* = 0·960) were not associated with having UIC < 100 µg/l. In the same way, households of WRA that did not use iodised salt (OR 1·200, 95 % CI 0·688, 2·095, *P* = 0·521) and the individuals who did not consume seafood in the past week (OR 1·346, 95 % CI 0·772, 2·348, *P* = 0·295) were not associated with having UIC < 100 µg/l (Table 4).

**Table 4.** Odds ratio (OR) of iodine deficiency (< 100 µg/l) in school-aged children (SAC) and women of reproductive age (WRA)

Variables	SAC					WRA				
	*n*	%	OR	95 % CI	*P*	*n*	%	OR	95 % CI	*P*
Granulated (iodised) salt used in the household*										
Not use or use other kinds of salt	31	19·9	1·175	0·358, 3·854	0·791	83	29·2	1·346	0·772, 2·348	0·295
Use	125	80·1	1	Ref		201	70·8	1	Ref	
Seafood consumption per week										
Never	72	42·4	1·024	0·407, 2·578	0·960	89	29·1	1·200	0·688, 2·095	0·521
≥ 1 times	98	57·6	1	Ref		217	70·9	1	Ref	

* Twenty-eight households (SAC, missing eighteen data; WRA, missing twenty-two data) did not provide salt samples.

All *P* values are given with logistic regression, with a 95 % CI.

Ref, reference value.

## Discussion

According to WHO guidelines for fortification of food-grade salt with iodine, salt iodisation is the desired strategy to prevent and control iodine deficiency disorders at the population level. At least 90 % of households, a representative population, should access an iodised salt with iodine content between 15 and 40 ppm^(17)^. In the present study, the majority (74·3 %) of the Karen households consumed granulated salt with a median iodine content of 26·64 (20·86–31·01) ppm, while 35·7 % of them consumed rock salt with a median iodine content of only 2·32 (0·52–3·19) ppm. Nevertheless, participants who used both rock and granulated salt in their households were not asked which type of salt they used more often. The current finding was consistent with the previous study in remote Australian Indigenous communities that iodised table salt was most commonly consumed (71·7 %) among all salt purchased^(18)^. More than 95 % of the Karen people regularly used salt in cooking rather than as a condiment and consume more than fish sauce, which is a rare iodine-fortified condiment that is popular with Thais of all ages, as shown in the 2017 Food Consumption Behavior Survey in Thailand^(19)^.

To determine iodine sufficiency, the median UIC of SAC (192 µg/l) and WRA (147 µg/l) in the Karen community indicated adequate iodine nutrition^(2)^. In addition, no more than 20 % of the SAC and WRA population should have a median UIC < 50 μg/l^(2)^; only 2 % of SAC and 9 % of WRA had values less than this cut-off. Our finding is consistent with the Iodine Global Network Global Scorecard 2021, which showed adequate iodine intake of Thai SAC (4). Interestingly, approximately one-fifth of SAC (20 %) and more than one-seventh of WRA (14 %) had excessive levels of median UIC (≥ 300 µg/l)^(2)^. Based on the Iodine Global Network Global Scorecard 2021, 11 out of 194 countries have shown excessive iodine intake in the SAC population (4). However, UIC assessment in spot urine samples in this study should be considered because it may be affected by several factors, such as hydration status and daily variation in iodine intake^(20,21)^. The study from Barloggio *et al.* stated that assessing thyroglobulin complementary with adjusting the UIC using urinary creatinine could strengthen the validity of the result rather than relying on UIC alone^(22)^. To estimate the daily iodine intakes in the population, the formula of the US Institute of Medicine has widely been used^(7,16,23)^. This study revealed that the daily iodine intakes of SAC (135 μg/d) and WRA (195 μg/d) were higher than the RDA for iodine^(2)^. Although the daily iodine intake of the participant group did not reach the international reference values for upper intakes of iodine^(24)^, the high level of iodine status in this Karen community is still a concern.

To determine the possible causes affecting the iodine status of the Karen people, the findings showed that the use of iodised salt and the frequency of seafood consumption per week were not associated with having UIC < 100 µg/l in both SAC and WRA. It indicates that the type of salt and seafood consumption are not good predictors of inadequate iodine intake in this Karen community. Other predictors of insufficient iodine status may be considered, such as salt storage, industrialised seasoning consumption and processed food consumption^(25,26)^. Besides considering insufficient iodine levels, excessive iodine intake should also be followed up. As findings, almost half of the SAC (47·7 %) and one-third of WRA (34·0 %) had UIC above the requirement. Adverse consequences of excessive iodine intake may activate hyperthyroidism, hypothyroidism, goitre and/or thyroid autoimmunity^(24,27)^. Accumulating iodine intake reaching excess levels may be caused by various sources, including iodised salt, drinking water, milk, certain seaweeds, dietary supplements containing iodine and processed foods and condiments composed of iodised salt^(28,29)^. All-source intake should be carefully considered^(24)^. Nevertheless, most people generally well-tolerate to excess iodine exposure or ingestion, except in some susceptible individuals^(30)^. Discretionary choice consumption among SAC in Tanzania can cause one-third of the children to have UIC > 500 µg/l. Most frequently consuming potato chips and fried cassava with discretionary salt added was likely caused by the excessive iodine intake in SAC^(31)^. Thus, a more elaborate and quantitative food frequency questionnaire or a 24-h dietary recall should be additionally applied to determine additional foods that are possible sources of excessive iodine consumption in further study. Although several limitations are discussed, the strengths of this study are also described. This is the first study that evaluates iodine sufficiency in the Pwo Karen communities in Thailand. Since most people in the communities speak and understand the Karen language more than the official Thai language, training local people, mostly village health volunteers, to be data collectors is advantageous because they comprehend their community context and understand the context of health surveillance and prevention. Moreover, they may play a key role in assisting the primary health care unit in monitoring the health of people in their communities in the future.

The findings affirm that the universal salt iodisation programme in Thailand is steady, as indicated by the majority consumption of iodised salt among the Indigenous population in remote areas. The strengths of the majority of iodised salt intake in this population should continue to be assured. However, iodine intake above the requirement of the Pwo Karen people should be closely monitored to determine the cause of this problem. These data should be confirmed in a larger population with an additional dietary assessment to ensure these marginalised people are receiving appropriate iodine intake, reducing the risk of many adverse health and nutrition consequences. In addition, the iodine deficiency disorder surveillance programme should continue to be actively extensive for all groups of people in the country.
